# Glycyrrhizin through liquorice intake modulates ACE2 and HMGB1 levels—A pilot study in healthy individuals with implications for COVID-19 and ARDS

**DOI:** 10.1371/journal.pone.0275181

**Published:** 2022-10-17

**Authors:** Felix Buder, Simina-Ramona Selejan, Mathias Hohl, Michael Kindermann, Christian Herr, Philipp M. Lepper, Robert Bals, Bernd Salzberger, Felix Mahfoud, Michael Böhm

**Affiliations:** 1 Department of Internal Medicine III—Cardiology, Angiology and Intensive Care Medicine, University Hospital, Saarland University, Homburg, Saar, Germany; 2 Department of Internal Medicine, Cardiology and Intensive Care Medicine, Caritas Hospital St. Theresia Saarbrücken, Saarbrücken, Germany; 3 Department of Internal Medicine V–Pulmonology, Allergology and Critical Care Medicine, University Hospital, Saarland University, Homburg, Saar, Germany; 4 Helmholtz Institute for Pharmaceutical Research Saarland (HIPS), Helmholtz Centre for Infection Research (HZI), Saarland University Campus, Saarbrücken, Germany; 5 Department for Infection Control and Infectious Diseases, University Hospital Regensburg, Regensburg, Germany; 6 Cape Heart Institute, Faculty of Health Sciences, University of Cape Town, Cape Town, South Africa; Medical Center - University of Freiburg, GERMANY

## Abstract

**Background:**

Glycyrrhizin, an active component of liquorice root extract, exhibits antiviral and immunomodulatory properties by direct inhibition of the pro-inflammatory alarmin HMGB1 (High-mobility group box 1).

**Objective:**

The aim of this study was to explore the role of liquorice intake on the viral entry receptor ACE2 (angiotensin-converting enzyme 2) and the immunoregulatory HMGB1 in healthy individuals and to explore HMGB1 expression in coronavirus disease 2019 (COVID-19) or non-COVID-19 in ARDS (acute respiratory distress syndrome patients).

**Material and methods:**

This study enrolled 43 individuals, including hospitalised patients with i) acute respiratory distress syndrome (ARDS) due to COVID-19 (n = 7) or other underlying causes (n = 12), ii) mild COVID-19 (n = 4) and iii) healthy volunteers (n = 20). Healthy individuals took 50 g of liquorice (containing 3% liquorice root extract) daily for 7 days, while blood samples were collected at baseline and on day 3 and 7. Changes in ACE2 and HMGB1 levels were determined by Western blot analysis and enzyme-linked immunosorbent assay, respectively. Additionally, HMGB1 levels were measured in hospitalised COVID-19 patients with mild disease or COVID-19 associated acute respiratory distress syndrome (ARDS) and compared with a non-COVID-19-ARDS group.

**Results:**

Liquorice intake significantly reduced after 7 days both cellular membranous ACE2 expression (-51% compared to baseline levels, p = 0.008) and plasma HMGB1 levels (-17% compared to baseline levels, p<0.001) in healthy individuals. Half of the individuals had a reduction in ACE2 levels of at least 30%. HMGB1 levels in patients with mild COVID-19 and ARDS patients with and without COVID-19 were significantly higher compared with those of healthy individuals (+317%, p = 0.002), but they were not different between COVID-19 and non-COVID-19 ARDS.

**Conclusions:**

Liquorice intake modulates ACE2 and HMGB1 levels in healthy individuals. HMGB1 is enhanced in mild COVID-19 and in ARDS with and without COVID-19, warranting evaluation of HMGB1 as a potential treatment target and glycyrrhizin, which is an active component of liquorice root extract, as a potential treatment in COVID-19 and non-COVID-19 respiratory disease.

## Introduction

Glycyrrhizin is an ingredient of liquorice and is in all probability the active agent of liquorice root extract [1–3]. During World War I, glycyrrhizin in form of liquorice was used to prevent coughs and colds [4]. It is also used in traditional Chinese medicine to treat respiratory infections [5, 6]. It has been hypothesized that glycyrrhizin could modulate the entry of SARS-CoV-2 (severe acute respiratory syndrome coronavirus type 2) and other viruses into the host cell by modulating the virus receptor ACE2 [7]. HMGB1 plays a significant role in the initiation of inflammation by promoting the expression of pro-inflammatory cytokines and activating immune cells [8, 9], and is therefore a potential biomarker and therapeutic target in situations with overwhelming immune response [10, 11]. This study aims to investigate i) the effects of glycyrrhizin through liquorice intake on cellular expression of ACE2 and HMGB1 plasma levels in healthy individuals after glycyrrhizin intake and ii) the regulation of HMGB1 in ARDS (acute respiratory distress syndrome) with or without COVID-19 and mild COVID-19.

## Material and methods

### Study design and treatment

We included healthy individuals without chronic disease or signs of respiratory infection (n = 20) and hospitalised patients (n = 23). The latter had either mild COVID-19 symptoms (n = 4), COVID-19 with ARDS (n = 7) or ARDS due to other underlying causes (n = 12). The demographics data are shown in Table 1. Healthy individuals were given 50 g of liquorice sweets (Black licorice wheels, Haribo GmbH & Co. KG) for 7 days. These contained the following ingredients: brown sugar syrup, all-purpose flour, glucose syrup, starch, liquorice root extract (3%), aroma, table salt, coating agents: beeswax white and yellow, carnauba wax. The liquorice dose corresponded to the recommendations of the German Federal Institute for Risk Assessment and the European Scientific Committee on Food, who consider 100 mg glycyrrhizin per day, equivalent to 50 g liquorice per day, to be uncritical [12, 13]. HMGB1 levels as well as electrolyte and blood pressure values were measured at baseline and on day 3 and 7, respectively. Of the 20 healthy individuals, 14 attended both follow-ups (day 3 and day 7). Additionally, peripheral blood mononuclear cells (PBMCs) were isolated from blood samples at baseline and day 7 and PBMC membranous ACE2 contents were determined at baseline and day 7. In hospitalised patients, the diagnosis of COVID-19 was based on the detection of SARS-CoV-2 nucleic acid and corresponding respiratory symptoms. Mild COVID-19 courses were defined as hospitalised patients treated on non-ICU wards and not requiring high-flow oxygen therapy. ARDS diagnosis was made using the Berlin criteria, which take clinical, radiological, respiratory and laboratory parameters into account [14]. COVID-19 patients with a severe course (COVID-19+ARDS) were treated on ICU and had an acute lung injury according the Berlin criteria. Peripheral blood was collected from all hospitalised patients and HMGB1 levels were measured. This study was approved by the local ethics committee (Nr 62/20 and Nr 105/12) in accordance with the Declaration of Helsinki. All participants gave written informed consent. If a patient was unable to consent the written consent of the legal representative was obtained.

**Table 1:** Patient characteristics.

Parameters	Healthy individuals	COVID-19 patients with mild course	ARDS patients with COVID-19	ARDS patients without COVID-19
**Number of patients**	20	4	7	12
**Sex**				
Female	10 (50.0%)	2 (50.0%)	1 (14.3%)	0 (0.0%)
**Age, years**				
Mean (SD)	40.8 (12.2)	80.0 (11.4)	59.9 (7.3)	68.8 (13.6)
Median (IQR)	40.0 (21.0)	83.0 (11.0)	58.0 (9.0)	68.5 (7.3)
**Age group, years**				
< 60	19 (95.0%)	0 (0.0%)	4 (57.1%)	1 (7.7%)
**Comorbidities**	n.a			
Hypertension		3 (75.0%)	3 (42.9%)	7 (58.3%)
Diabetes		2 (50.0%)	0 (0.0%)	4 (33.3%)
Cardiovascular disease		3 (75.0%)	1 (14.3%)	5 (41.7%)
Chronic pulmonary disease		0 (0.0%)	0 (0.0%)	3 (25.0%)
**Obesity category by BMI, kg/m^2^**	n.a			
No Obesity (<25)		1 (33.3%)	2 (28.6%)	3 (25.0%)
Overweight (≥25 and <30)		1 (33.3%)	3 (42.9%)	4 (33.3%)
Class 1 Obesity (≥30 and <35)		1 (33.3%)	2 (28.6%)	2 (16.7%)
Class 2 Obesity (≥35 and <40)		0 (0.0%)	0 (0.0%)	0 (8.0%)
Class 3 Obesity (≥40)		0 (0.0%)	0 (0.0%)	2 (16.7%)
Unknown		1 (33.3%)	0 (0.0%)	1 (8.3%)
**Length of hospital stay, days**	n.a			
Mean (SD)		19.0 (16.6)	41.1 (16.7)	33.3 (19.9)
Median (IQR)		14.0 (9.5)	45.0 (17.5)	28.5 (32.8)
**Deaths**	n.a	0 (0.0%)	0 (0.0%)	3 (25.0%)

### Western blot analysis

From 8 of the healthy study subjects, PBMCs were isolated from venous blood by Ficoll density gradient centrifugation (Biochrom). After washing the cells with ice-cold PBS (pH 7.4), the cell pellets were resuspended in hypotonic buffer (5 mmol/L Tris, 1 mmol/L EDTA, 5 mmol/L MgCl_2_, pH 8.0, 1 mmol/L PMSF, 1 mmol/L leupeptin, 5 μg/ml aprotinin) at 4°C for 15 min. To obtain a "cytosolic" (supernatant) and a "membrane" fraction (pellet), the suspension was subjected to 100000 g ultracentrifugation (1 h, 4°C). The membrane fraction obtained was resuspended in 100 μl hypotonic buffer containing the protease inhibitors described above. Protein concentrations were determined using the method of Lowry [15] and samples of the membrane fraction were analysed for ACE2 by Western blot. 50 μg of protein were denatured (95°C for 5min), separated on 10% SDS-PAGE and transferred to nitrocellulose membranes (Protran, Schleicher & Schuell GmbH, Dassel, Germany) by semidry electrophoretic blotting. Anti-ACE2 (ab15348, 1:1000, Abcam, Cambridge, UK) was used for primary antibody and anti-rabbit (#172–1019, Bio-Rad Laboratories, Inc., USA) for secondary antibody. Equal loading of total protein (50 μg/lane) was controlled by Ponceau red staining. Data are reported as integrated optical density (IOD) normalised to a control sample and to Ponceau red unless otherwise stated. Proteins were visualized by enhanced chemiluminescence (ECL) following the manufacturer’s protocol (Amersham Pharmacia Biotech, Freiburg, Germany) and analysed by the “LabWorks 4.6” Software (LabWorks Image Acquisition and Analysis Software, UVP BioImaging Systems, Cambridge, UK).

### ELISA

Peripheral blood was collected and plasma concentrations of HMGB1 (human HMGB1 ELISA kit, Cat#EKF57253, Biomatik, USA) were measured by ELISA according to the manufacturer’s instructions.

### Statistical analysis

Data were tested for normal distribution. When comparing ≥3 groups, a Friedman analysis with Dunn’s post-hoc correction was performed for paired groups, and a Kruskal-Wallis ANOVA with Dunn’s post-hoc test correction was performed for unpaired data samples. A Wilcoxon test was used for paired groups. Statistical significance was set at an alpha level of p<0.05. Data were analysed using GraphPad Prism version 8.4.3 (GraphPad Software Inc., CA, USA) and are shown as median with interquartile range (IQR).

## Results

### Liquorice intake reduces ACE2 levels in healthy individuals

**Fig 1:**
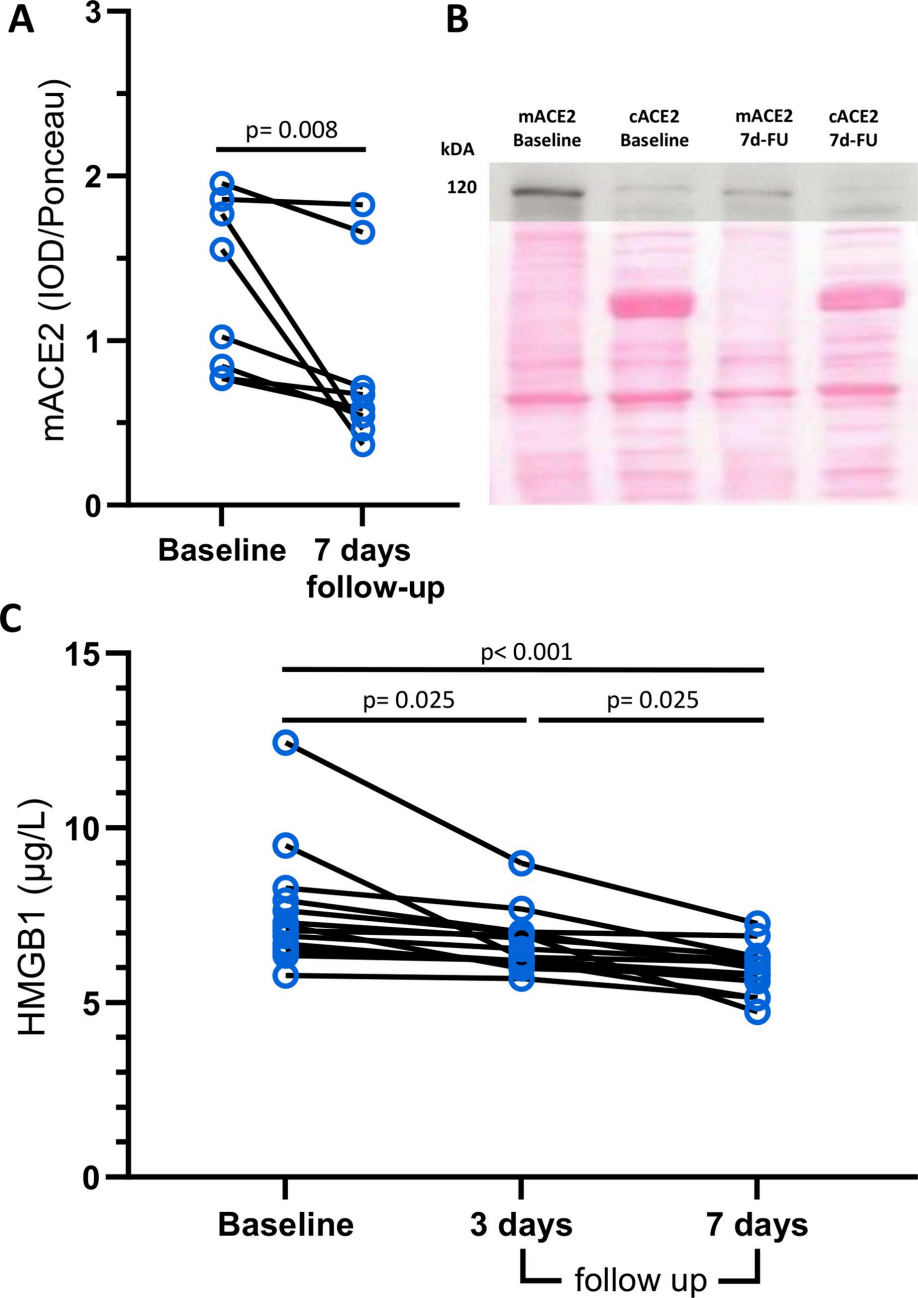
Modulation of mACE2 and HMGB1 levels after liquorice intake in healthy individuals. *A*, PBMC mACE2 levels before and 7 days after liquorice ingestion. *B*, Representative ACE2 Western blot (upper panel) of membraneous (mACE2) and cytosolic (cACE2) fractions from PBMCs from one healthy volunteer at baseline and after 7 days (7d-FU) of liquorice ingestion. The corresponding Ponceau Red strip showing total protein loading is presented below (lower panel). *C*, Plasma HMGB1 levels before as well as 3 and 7 days after liquorice ingestion. Wilcoxon test and Friedman analysis with Dunn’s post-hoc test were performed when comparing 2 groups and 3 groups respectively. Data are shown as median±IQR. Abbreviation: mACE2, membranous ACE2; HMGB1, high-mobility group box 1 protein; PBMC, peripheral blood mononuclear cell; IQR, interquartile range.

Median age of the healthy individuals (10 females) was 40.0 years (IQR, 31.3–52.3). Median baseline systolic and diastolic blood pressure were 126 mmHg (IQR, 123–130) and 75 mmHg (IQR, 74–81), respectively. No significant differences in blood pressure could be found compared with baseline, neither at day 3 (118 mmHg (IQR 116–130), p = 0.18 and 74 mmHg (IQR 73–83), p = 0.82, respectively) nor at day 7 (130 mmHg (IQR 127–132), p = 0.30 and 79 mmHg (IQR 74–82), p = 0.72, respectively). In 8 healthy individuals, PBMCs were isolated from peripheral blood at baseline and after 7 days. After 7 days of liquorice intake, significantly lower membranous levels of ACE2 were observed compared with baseline (1.3 (IQR 0.8–1.8 vs. 0.6 (IQR 0.5–1.4) IOD/Ponceau, p = 0.008) (Fig 1A). Of note, all healthy individuals showed a reduction in ACE2 levels after liquorice intake, and in half of the healthy individuals, ACE2 levels were reduced by at least 30%, with 2 of them measuring a drop greater than 70%.

### Liquorice intake reduces HMGB1 levels in healthy individuals

After liquorice intake, both on day 3 and day 7, HMGB1 levels were significantly lower compared with baseline (6.4 (IQR 6.2–7.0) vs. 7.1 (IQR 6.6–8.0) μg/L on day 3, p = 0.025 and 5.9 (IQR 5.5–6.3) vs. 7.1 (IQR 6.6–8.0) μg/L on day 7, p<0.001, respectively) (Fig 1C). In all individuals, the HMGB1 levels decreased after 3 days and were lowest after 7 days. In half of the individuals, HMGB1 levels were reduced by at least 15%.

### Enhanced HMGB1 levels in ARDS patients

**Fig 2:**
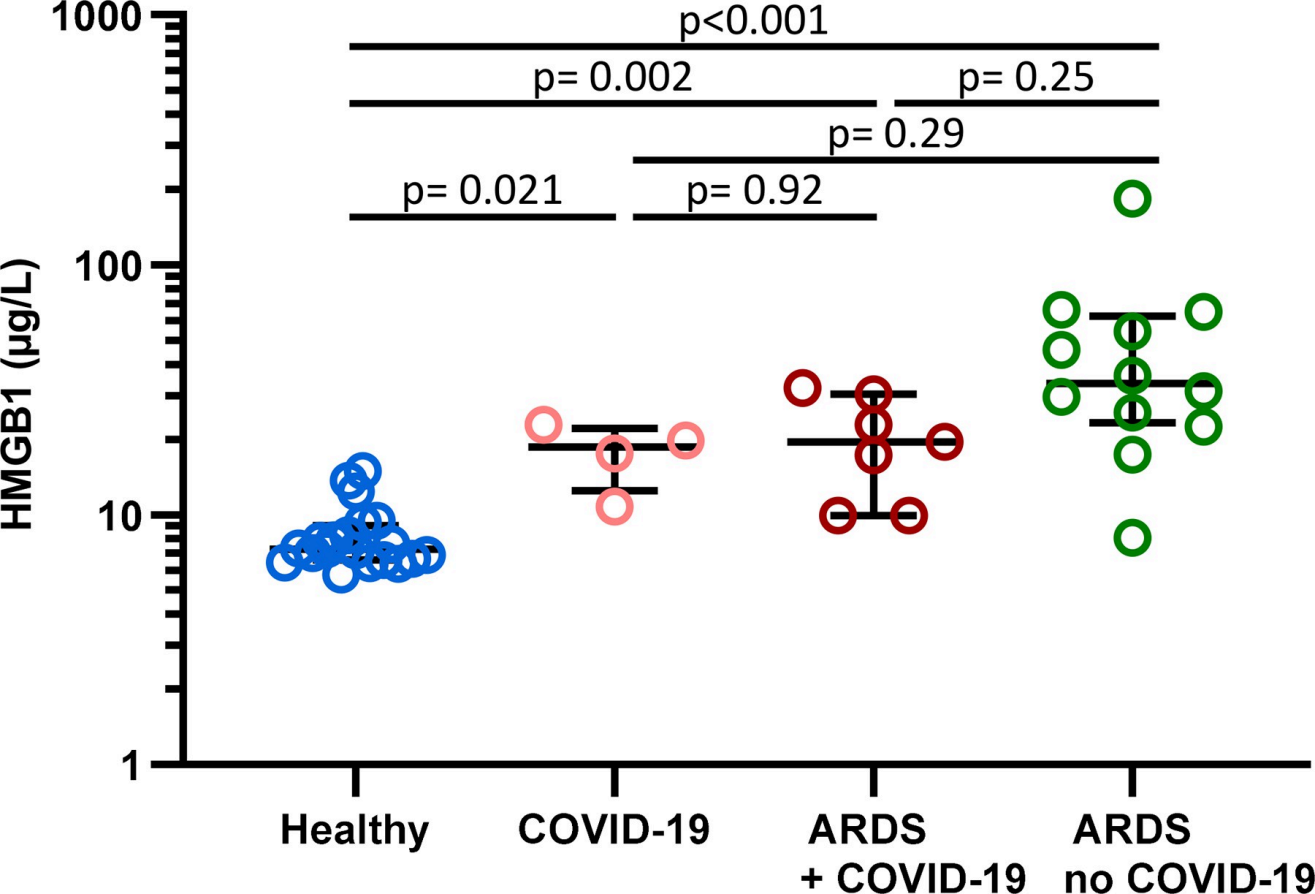
HMGB1 levels in healthy individuals, in COVID-19 patients with mild or severe course (ARDS) and in ARDS patients without COVID-19. HMGB1 levels were evaluated in healthy individuals (n = 20), COVID-19 patients with a mild (n = 4) course and ARDS (n = 7) respectively, as well as ARDS patients without COVID-19 (n = 12). Data are shown as median±IQR. Kruskal-Wallis ANOVA with Dunn’s post-hoc test was performed. Abbreviation: ARDS, acute respiratory distress syndrome; COVID-19, Coronavirus disease 2019; HMGB1, high-mobility group box 1 protein; IQR, interquartile range.

A total of 23 hospitalised patients (3 females) at a median age of 66.0 years (IQR, 63.0–74.5) were enrolled in the study, out of which 11 patients had laboratory confirmed SARS-CoV-2 infection with COVID-19 typical symptoms, including 7 patients with ARDS. Another 12 patients had ARDS with different underlying causes other than COVID-19, i.e. due to systemic causes (n = 3) or due to primary damage to the lungs (n = 9). HMGB1 levels between ARDS with and without COVID-19 were not significantly different (p = 0.25), with a median of 19.6 μg/L (IQR, 10.0 to 30.3) and 33.6 μg/L (IQR, 23.3 to 62.4), respectively (Fig 2). These cohorts showed 2.5- and 5.9-fold higher HMGB1 levels than the baseline values of healthy individuals (p = 0.002 and p<0.001, respectively). The ARDS without COVID-19 cohort showed a wide dispersion of HMGB1 levels, in that concentrations differed inter-individually up to a factor of 23. COVID-19 patients with a mild course had significantly higher HMGB1 levels compared to healthy individuals before treatment (18.7 (IQR 12.5–22.1) vs. 7.3 (IQR 6.6–9.0) μg/L, p = 0.021), but showed no significant difference from either ARDS cohort (ARDS with COVID-19 19.6 μg/L (IQR 10.0–30.2), p = 0.92 and ARDS without COVID-19 33.6 μg/L (IQR 23.3–62.4), p = 0.29).

## Discussion

The key findings of the present study are: i) Plasma HMGB1 levels and cellular ACE2 were modulated by liquorice administration in healthy individuals, and ii) HMGB1 levels in severe lung injury were increased compared with healthy individuals but not different in COVID-19 ARDS versus non-COVID-19 ARDS.

The active agent of liquorice root extract is in all probability glycyrrhizin [1, 3]. The efficacy of glycyrrhizin in respiratory tract infections could be partially based on its ability to prevent viruses from ACE2 mediated cell entry. The transmembrane enzyme ACE2 recently gained attention as it was identified as the essential receptor for SARS-CoV-2 cell entry [16]. In vitro, glycyrrhizin inhibited the replication of SARS-CoV [17] and SARS-CoV-2 [18], both of which belong to the genera of betacoronaviruses and use the same viral entry. Therefore, apart from the direct effects on viral replication the glycyrrhizin effects could also be due to the regulation of cellular ACE2. Furthermore, in a rat model in which 24 male Sprague-Dawley rats were treated with a glycyrrhizin extract for 2 weeks, significantly lower ACE2 gene expression was found in the small intestine compared to the control group [7]. In silico, i.e. using computer simulation, it was shown that glycyrrhizin has the ability to bind to ACE2 itself [19], implying that viral entry in host cells could be blocked, and thereby SARS-CoV-2 infections could be prevented. Data from our study extend those previous findings, by showing that 7-day liquorice intake resulted in reduction of ACE2 expression in PBMCs by up to 51%.

Glycyrrhizin is furthermore associated with anti-inflammatory activity by reducing ROS generation in neutrophils [20] and downregulating cytokines [21, 22]. HMGB1, which modulates cytokine release and leads to overwhelming pro-inflammatory states [9, 23], may play a key role as glycyrrhizin binds to it [22, 24] and inhibits the phosphorylation of HMGB1 [25], rendering it less active and less detrimental. In cellular models and rodent models, glycyrrhizin administration resulted in a significant decrease in HMGB1 levels [22, 26]. Our study extends these findings to humans, showing reduced HMGB1 levels after liquorice intake in healthy individuals. To our knowledge, this is the first study on the inhibitory effect of glycyrrhizin on HMGB1 in humans. The most pronounced effects were seen in individuals with the highest baseline HMGB1 levels. In addition a direct interaction of HMGB1 and ACE2 expression has been suggested [27]. Our data show that expressions of both proteins—HMGB1 and ACE2—can be decreased after liquorice intake. This congruent relationship between HMGB1 and ACE2 is consistent with the observation that ACE2 increases after treatment with HMGB1 in alveolar epithelial cells [27]. The effects are significant although individuals were given low doses of liquorice (50g per day) in our study, which are considered to be safe according to the recommendations of the German Federal Institute for Risk Assessment and European Scientific Committee on Food [12, 13]. High doses of liquorice can cause aldosterone-like effects with increases in blood pressure and decreases in potassium levels [28]. Due to the moderate dosage of liquorice used herein, we did not observe any adverse effects during the study.

Extracellular HMGB1 is a key damage-associated molecular pattern molecule (DAMP) released by infected or damaged cells as well as activated immune cells and promotes the expression of pro-inflammatory cytokines as well as macrophages activation [8, 9]. While HMGB1 release is immediate in necrotic cell death, active HMGB1 release in inflammatory diseases plateaus after 16–32 hrs [29]. In addition to its key pathophysiological role in the initiation of inflammation, it is, therefore, also a potential biomarker and therapeutic target in situations with an overwhelming immune response [10, 11]. Our results are consistent with previous studies showing increased HMGB1 levels in ARDS patients [11, 30]. HMGB1 has been shown to be increased by a factor of 5 in severe COVID-19 courses compared to mild courses and is therefore discussed as a potential biomarker for severe COVID-19 courses [27]. Our data do not permit differentiation with regard to disease severity, as HMGB1 levels are not significantly different in COVID-19 patients with mild courses or with ARDS in the present study. One possible explanation is the modest number of patients included in both COVID-19 groups and especially in the “mild-course” COVID-19-group, which limits discrimination of disease severity. Also, in the “mild-course” COVID-19 group patients with diabetes mellitus were overrepresented (50%) and HMGB1 levels are known to be increased in diabetes mellitus [31]. Therefore, in the “mild-course” COVID-19 group, HMGB1 levels could rather be a marker of diabetes mellitus than COVID-19 disease severity. Other groups have shown that HMGB1 can be drastically increased in severe COVID-19 courses compared to mild courses [27], but our study was not powered for that scope.

There is evidence that glycyrrhizin may protect against respiratory tract infections, including coronaviruses, flaviviruses and influenza virus infections [32–34]. Recently published data provide insight on glycyrrhizin effects in ARDS [35]. Mice with ARDS had significantly reduced HMGB1 levels after glycyrrhizin administration, while lung function improved [35]. In particular, viral polymerase activity was inhibited after administration of glycyrrhizin [32]. Glycyrrhizin was also effective in inhibiting the replication of parainfluenza viruses by reducing genome RNA and protein expression, including fusion and hemagglutinin neuraminidase proteins [36]. Interestingly, the replication of SARS which belongs to the genera of beta-coronaviruses, like SARS-CoV-2, was also effectively inhibited by glycyrrhizin [17, 37]. Accordingly, a recent study revealed that non-toxic concentrations of glycyrrhizin inhibited the replication of SARS-CoV-2 in vivo by blocking the main protease Mpro of SARS-CoV-2 [18].

Initial results of a phase 3 study provide first clinical data on glycyrrhizin use in 120 ICU patients with COVID-19 [38]. Both groups received state of the art treatment (including remdesivir and glucocorticoids), while the intervention group was additionally administered a herbal-based combination of 5 agents, including glycyrrhizin, for up to 2 weeks. Randomization to the treatment group was associated with a significantly lower risk of mortality (odds ratio, 0.14; 95% CI, 0.05–0.32, p<0.001) compared with the control group. The data could help to enlighten the therapeutic potential of glycyrrhizin in COVID-19, although the number of patients included is still small and glycyrrhizin was not used alone but as part of a herbal combination treatment.

Some limitations of our study need to be acknowledged. The number of hospitalised patients was limited and in this pilot study we did not investigate the modulation of ACE2 and HMGB1 after glycyrrhizin administration in diseased patients, however, our data sustain the hypothesis of a potential use of glycyrrhizin in ARDS with and without viral infection. Further studies are needed to clarify the role of HMGB1 along the course of viral infections from mild viral diseases to ARDS.

## Conclusions

In summary, intake of liquorice reduces ACE2 expression and HMGB1 levels in healthy humans. The latter of which is increased in ARDS patients with and without COVID-19 as well as in mild COVID-19. The discussed mechanisms of action of glycyrrhizin through liquorice intake, namely the inhibition of ACE2 and HMGB1, both known to facilitate SARS-CoV-2 cellular entry and inflammation, respectively, and the efficacy of glycyrrhizin in several viral diseases, identify glycyrrhizin as a potential therapeutic in COVID-19 but also in other respiratory tract diseases. Glycyrrhizin is widely available, cheap, and safe. The preventive usage as well as combinations with existing therapeutic drugs could be promising for COVID-19, but need to be explored in further clinical trials.

## Supporting information

S1 Raw images (PDF)

S1 File (DOCX)
