# Supplementary material for: Glycyrrhizin through liquorice intake modulates ACE2 and HMGB1 levels—A pilot study in healthy individuals with implications for COVID-19 and ARDS
Source: PLoS One. 2022 Oct 17;17(10):e0275181. doi: 10.1371/journal.pone.0275181 (PMC9576069; doi:10.1371/journal.pone.0275181)

Images underlying gel results for Figure 1 a

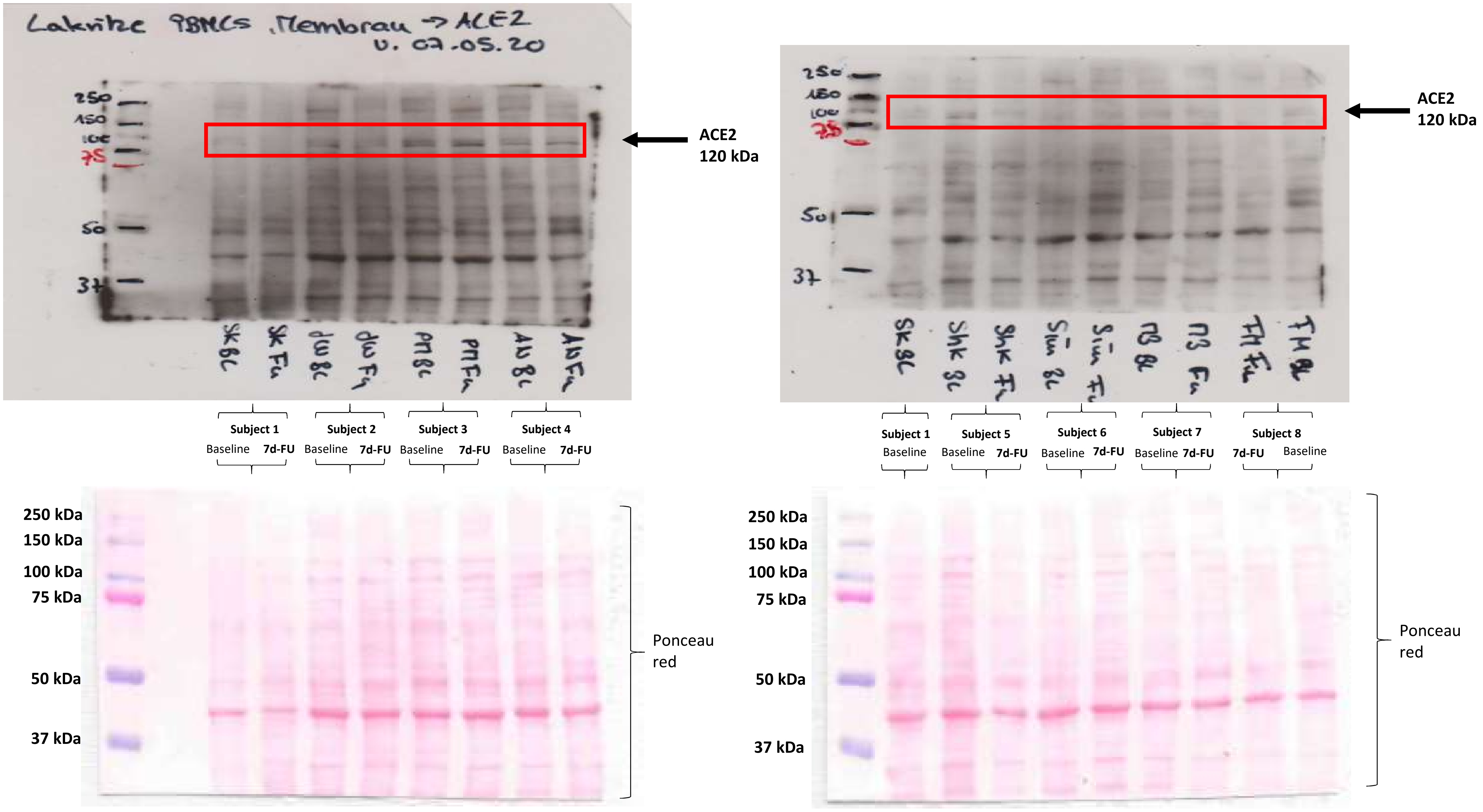

Original gel underlying Figure 1 b

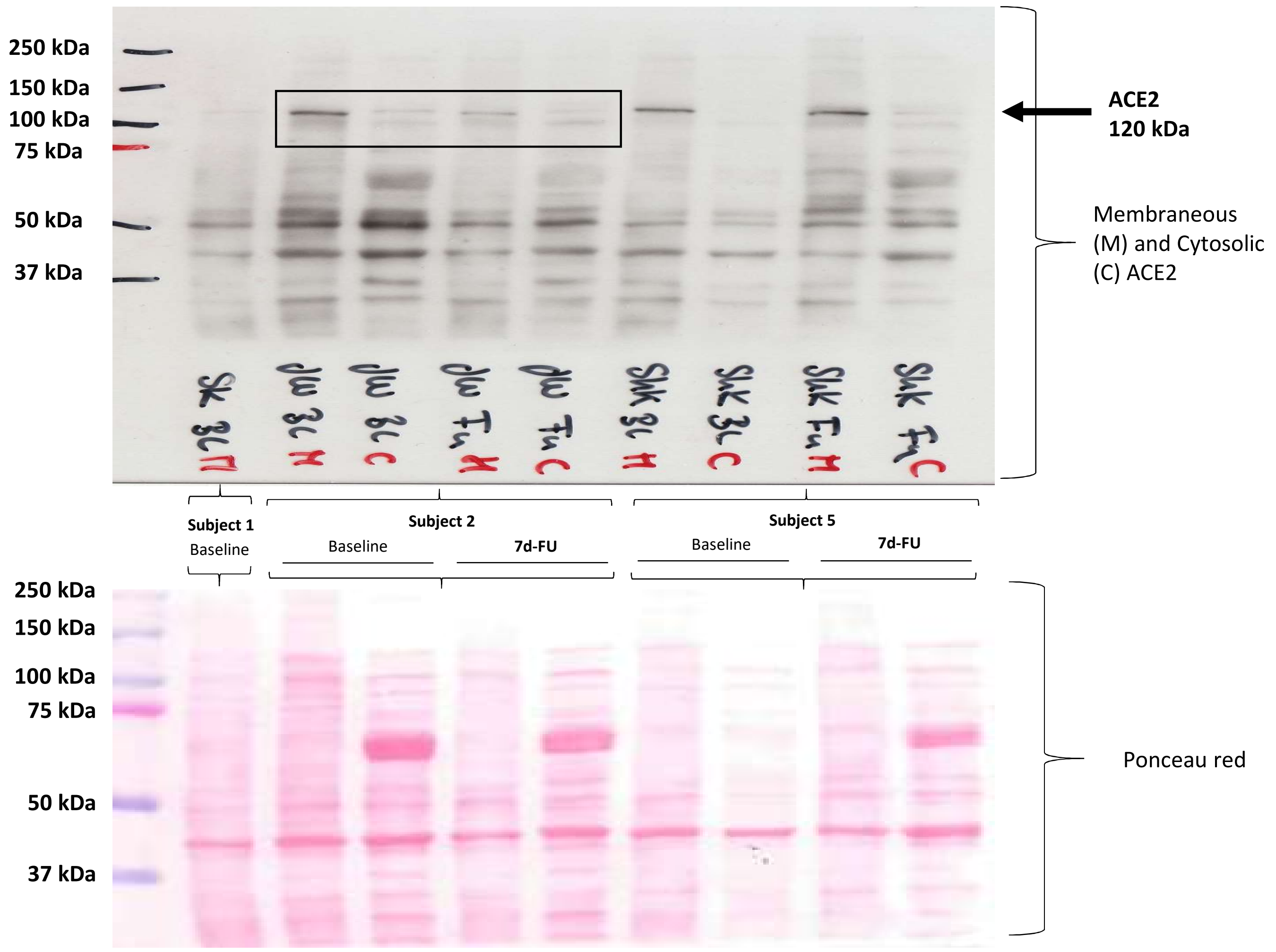

Supplement: S1 Raw images — (PDF) [file pone.0275181.s001.pdf]
