# Supplementary material for: Glycyrrhizin through liquorice intake modulates ACE2 and HMGB1 levels—A pilot study in healthy individuals with implications for COVID-19 and ARDS
Source: PLoS One. 2022 Oct 17;17(10):e0275181. doi: 10.1371/journal.pone.0275181 (PMC9576069; doi:10.1371/journal.pone.0275181)
Supplement: S1 File — (DOCX) [file pone.0275181.s002.docx]

**Table 1. Characteristics of ARDS patients without COVID-19**

| Patient number | Aetiology | Comorbidities | Obesity category by BMI, kg/m² | Length of hospital stay, days |
| --- | --- | --- | --- | --- |
| 1 | Sepsis due to pneumonia | Hypertension DM | 27,8 | 75 |
| 2 | Aspiration pneumonia | CVD | 27,3 | 29 |
| 3 | Pneumonia | Hypertension DM CVD | 27,8 | 14 |
| 4 | Pneumonia | Hypertension CVD | 23,4 | 53 |
| 5 | Sepsis | DM Pulm. disease | 28,4 | 39 |
| 6 | Pneumonia | - | 58,1 | 49 |
| 7 | Pneumonia | - |  | 10 |
| 8 | Pulmonary sarcoidosis | Hypertension DM | 21,6 | 48 |
| 9 | Sepsis | . | 41,0 | 16 |
| 10 | Pneumonia | Hypertension CVD | 30,9 | 26 |
| 11 | Pneumonia | Hypertension  Pulm. disease | 30,8 | 13 |
| 12 | Pneumonia | Hypertension CVD  Pulm. disease | 21,7 | 28 |

Abbreviations: BMI, body mass index; DM, diabetes mellitus; CVD, cardiovascular disease, pulm. disease; chronic pulmonary disease.

**Table 2. Characteristics of ARDS patients with COVID-19**

| Patient number | Comorbidities | Obesity category by BMI, kg/m² | Length of hospital stay, days |
| --- | --- | --- | --- |
| 1 | Hypertension  CVD | 30,5 | 60 |
| 2 | - | 27,0 | 36 |
| 3 | Hypertension | 32,7 | 33 |
| 4 | Hypertension | 29,6 | 50 |
| 5 | - | 24,0 | 54 |
| 6 | - | 24,0 | 45 |
| 7 | - | 25,9 | 10 |

Abbreviations: BMI, body mass index; CVD, cardiovascular disease.

**Table 3. Characteristics of ARDS patients with mild course.**

| Patient number | Comorbidities | Obesity category by BMI, kg/m² | Length of hospital stay, days |
| --- | --- | --- | --- |
| 1 | Hypertension | 29,3 | 5 |
| 2 | Hypertension  DM  CVD | 34,9 | 14 |
| 3 | Hypertension  CVD | 17,0 | 43 |
| 4 | DM  CVD | NA | 14 |

Abbreviations: BMI, body mass index; NA, not available; DM, diabetes mellitus; CVD, cardiovascular disease.
